# Supplementary material for: Are carbon emissions trading and green financial instruments synergistic? -Comprehensive quantitative research based on content analysis
Source: PLoS One. 2024 Mar 7;19(3):e0298601. doi: 10.1371/journal.pone.0298601 (PMC10919601; doi:10.1371/journal.pone.0298601)
Supplement: S1 Data — (ZIP) [file pone.0298601.s001.zip › Data/地级市Z_F工作报告.zip!/░▓╗╒/╦▐╓▌╩╨/└┤╫╘╣1⁄2╓┌║┼í╛┬φ┐╦╩2╛▌═°í┐_2019.docx]

2019年1月9日在宿州市第五届人民代表大会第二次会议上

市长　杨　军

各位代表：

现在，我代表市人民政府，向大会报告政府工作，请予审议，并请市政协委员和其他列席人员提出意见。

一、2018年工作回顾

2018年是改革开放40周年，也是本届政府的开局之年。一年来，面对复杂多变的经济形势，在省委、省政府和市委坚强领导下，全市上下深入学习贯彻习近平新时代中国特色社会主义思想和党的十九大精神，坚持稳中求进工作总基调，持续深化供给侧结构性改革，对标高质量发展要求，深入实施五大发展行动计划，全力打好三大攻坚战，奋力推进改革发展，较好地完成了市五届人大一次会议确定的主要目标任务，现代化五大发展美好宿州建设呈现新局面。

初步预计，全市生产总值增长8.5%；财政收入175.8亿元，增长12.3%；固定资产投资增长15.5%；社会消费品零售总额增长13%；城镇、农村常住居民人均可支配收入分别增长8.7%、10%；主要经济指标增速全省靠前，三次产业结构持续优化，全市经济运行保持总体平稳、进中向好的态势。

一年来，我们重点抓了以下六个方面工作：

一是突出创新驱动，产业结构持续优化。新型工业化持续推进。坚持工业强市战略不动摇，贯彻落实省促进经济高质量发展“30条”、促进民营经济发展“30条”等系列政策，工业总量加速扩张、质量明显提升。深入实施“全创改”“三重一创”，全力建设战略性新兴产业基地，规模以上工业增加值增长10.1%；战略性新兴产业产值、高新技术产业增加值分别增长20.1%、27.6%，占规上工业比重显著提升。实施“3111”工程和重点技改“1525”行动计划，县区、园区首位产业产值增长19.4%，技改投资增长44.8%，产业发展的体量更大、成色更足。区域发展齐头并进。宿州经开区生物医药科技园原料药制造项目入选省重大新兴产业工程。绿地科技产业园项目落户宿马园区。市高新区、砀山经开区去筹转正，市高新区数字小镇获批省级特色小镇。鞋城在中国轻工业产业集聚区综合评比中名列前茅。萧县获评全国县域经济投资潜力百强县，埇桥区获评全国投资潜力百强区、连续5年被授予全省“发展民营经济先进县区”称号。张江萧县高科技园创建沪皖合作示范园区被列入长三角地区合作要点。泗涂现代产业园在全省县域南北合作共建园区年度考核中排名首位。符离镇获评全国综合实力千强镇。宿州百丽鞋业荣登2018安徽省民营企业纳税百强、营收百强。项目建设提速提效。高质量落实“四督四保”制度，开展重点项目推进“百日会战”，607个省亿元以上重点项目完成投资680亿元，泰盛纸业、宿州会展中心等项目开工建设，新亚电子、绿色家居产业园家居生产等项目加快推进，钱营孜低热值煤电厂、紫金石墨烯复合薄膜产业化基地等项目竣工投产。质量品牌提升工程成效明显，新增安徽名牌产品16个。现代服务业有效提升。服务业增加值增长10.3%。新增贷款300亿元、增长25%，任务完成率全省第一；余额存贷比68%；新增存贷比110%，高于全省28个百分点。成功创建省级服务业集聚区、集聚示范园区各1家，宿州吾悦广场等项目开工建设。新汴河景区创建国家4A级景区进入公示阶段。砀山县、埇桥区获评全省电子商务进农村全覆盖工作先进县区。新增国家级服务标准化试点单位1家、省级3家。农业现代化加快发展。农业产业化水平稳居全省第一方阵，全市规模以上农产品加工产值占全省总产值的9.7%，农业产业化联合体发展到283家、位居全省第一。发端于宿州的农业产业化联合体模式写入2018年中央一号文件。农村“三变”改革已开展375个村、完成改革300个村。“三品一标”认证企业200家、产品289个。埇桥区荣获“中国烧鸡之乡”称号，萧县葡萄、泗县大路口山芋获批国家地理标志保护产品。

二是突出精准扶贫，脱贫攻坚成绩斐然。脱贫攻坚考核跃居全省第一方阵前列，打赢了翻身仗。围绕“1234+N”工作思路，全市统筹用于扶贫的财政资金达30.56亿元，“春夏秋冬”四季攻势全面收官，顺利完成115个贫困村出列、9.33万贫困人口脱贫的年度目标，全市贫困发生率降至1.22%，三县一区退出有望，脱贫攻坚取得决定性进展。扎实开展“重精准、补短板、促攻坚”专项整改行动、“七个不落、一个不少”大排查。创造性实施“四带一自”产业扶贫、“三有一网”点位扶贫等模式，到村产业项目覆盖率100%，实施特色种养业扶贫到户项目22.37万个，经营主体带动贫困户17.59万户。拓展实现所有学段、公办民办学校、家庭经济困难学生资助全覆盖。全面落实“351”“180”健康脱贫政策，拓展实施“两类人群”保障政策，创新实施“三类人员”集中供养，全市贫困人口家庭医生签约实现全覆盖。易地扶贫搬迁工程圆满收官，6576人全部搬迁入住，完成四类重点对象和边缘户危房改造31873户。新改建农村公路10451.6公里，全市基本实现自然村通硬化路。安全饮水工程自来水到户率实现贫困村全覆盖，非贫困村覆盖90%以上。农网供电全面升级，行政村实现光纤接入全覆盖。基层基本公共服务八大领域19项指标均达到或高于全省平均水平。我市在全国驻村帮扶工作座谈会上作经验介绍，荣获2018年度全国脱贫攻坚组织创新奖，涌现出曾翙翔等一批先进典型。创新实施“一自三合”扶贫小额信贷模式，全国金融扶贫现场培训在灵璧县举办。泗县健康脱贫工作受到国家卫健委和国务院扶贫办通报表彰。全省贫困县高质量退出现场推进会在泗县召开。

三是突出污染防治，环境保护克难奋进。面对污染防治的严峻形势，全市上下痛下决心、勠力同心，全面打响蓝天、碧水、净土“三大保卫战”，中央、省环保督察反馈问题整改有效推进，污染防治攻坚战取得阶段性成效。围绕“五控”工作，扎实开展大气污染防治百日攻坚行动，PM2.5平均浓度57.8微克/立方米、下降17.1%，下降率全省第四；空气优良天数比例60.3%、提高9.5个百分点。开展“洁净宿州”行动，划定高污染燃料禁燃区，城区全面禁售禁放烟花爆竹，加大重点区域机动车、非道路移动机械污染治理力度，全面落实建筑工地扬尘防治“六个百分百”。主城区重点区域煤改气全面完成，“散乱污”企业排查整治有效推进，282台燃煤小锅炉有279台完成清洁能源替代，192家砖瓦窑厂关闭淘汰并拆除到位，完成156个关停矿山生态修复。精准实施水污染治理，河长制、湖长制全面推行，4个国控考核断面水质达到或优于水质控制目标。启动主城区污水治理三年攻坚战，改造完成主城区6条道路污水管网、291处雨污合流管网截污点，11条黑臭水体治理初见成效，建成生活污水集中处理厂6座、工业园区集中污水处理厂11座，汴北污水处理厂投入运营，成功争获全国首批黑臭水体治理示范城市。全面落实“土十条”，扎实推进土壤污染治理与修复。深入开展“清废行动”，290余个固体废物污染问题得到解决，遗患多年的灵璧闵庄垃圾场变身为惠民利民的休闲公园。市县乡村四级林长制工作体系逐步健全。持续推进农村环境综合整治，禁养区内养殖场实施了关闭或搬迁，规模养殖场粪污处理设施装备配套率达90.2%，秸秆综合利用率、畜禽养殖废弃物资源化利用率分别达91.4%、81%。单位GDP能耗下降超额完成省下达目标任务。

四是突出统筹联动，城乡面貌显著改观。高度重视规划引领，新一轮城市总体规划修编完成，土地利用总体规划加快修编，专项规划进一步完善。中心城区实施四大类120项重点工程，完成投资150亿元。银河一路东西延伸工程、宿州火车站综合改造、宿州民航机场等项目前期工作加快推进，新汴河航道整治工程开工建设，符离大道、宿淮快速通道、G206北段等正在建设，西外环部分路段、拂晓大道北延建成通车，6条市政道路白改黑工程全面完成，市汽车客运中心站投入运营，智慧交通建设取得显著成效。“五馆一中心”部分竣工验收，春节前可投入使用。埇桥独立工矿区入列国家独立工矿区改造搬迁试点。全力推动全国文明城市创建，扎实开展“十项提升行动”，长期困扰市民日常生活的区域供水紧张、管线私拉乱接、车辆乱停乱放、交通混乱拥堵等一批身边难题正逐步得到解决。大力推进“两治三改”，中心城区完成征迁228万平方米、总量创出新高，安置房建设新开工43577套、基本建成11730套，开工率、基本建成率居全省前列。“555”工程加快实施，四个县建设重点工程100项，完成投资180.5亿元。乡村振兴战略开局良好，美丽乡村建设走在皖北前列，泗县彭铺村获得中国十大最美乡村、全国百佳乡村旅游目的地荣誉称号。在全省率先实施的城乡建设用地增减挂三年提升行动开局顺利，完成拆迁3.68万亩、复垦3.3万亩、节余指标交易3956亩，探索出了生态型增减挂的“宿州路径”。“四好农村路”建设深入实施。农村环境整治“三大革命”扎实推进，农村垃圾治理基本实现市场化，完成改厕13.4万户，建成乡镇政府驻地污水处理设施45个，泗县入列全省农村人居环境整治三年行动示范县。砀山县入选全省唯一的全国经济林产业区域特色品牌建设试点。萧县龙城镇、埇桥区闵祠村等1镇5村入选省第六批千年古镇、古村落。我市作为全省唯一国家级建设用地二级市场试点市，高质量通过自然资源部验收。顺利通过国家节水型城市创建验收、国家园林城市复核。

五是突出深化改革，开放发展更具活力。“破、立、降”全面发力，清理“僵尸企业”53家，非住宅商品房库存去化周期低于全省平均水平。“放管服”改革纵深推进，“两难两多一长”有效整治，政务服务“一网、一门、一次”改革扎实推进，在政务服务分办秒办、帮办代办等多项便民利企举措上采取“一招鲜”，推动开办企业“一窗办”，企业开办时间压缩至3个工作日以内，民营企业获得感明显增强。“四送一服”双千工程深入开展，“四最”营商环境持续优化，城市信用排名迈入全国第一方阵，全年新登记各类市场主体5.7万户、增长24.4%。国企改革扎实有序，市属国有企业完成产权划转重组34家、公司制改制17家。户籍制度改革深入实施，农业转移人口落户城镇10万余人。全面完成存量政府债务置换、减少政府债务利息支出13亿元，政府债务余额低于省政府核定限额，宿州农商行不良贷款率下降10个百分点，风险总体可控。金融改革稳步推进，“新三板”和省股权交易中心挂牌企业新增72家，全市投资基金规模达205亿元，发放续贷过桥资金等创新业务贷款110亿元。深化商事制度、财税体制、投融资体制、公共资源交易管理体制等重点领域和关键环节改革，127项改革任务高效落实。宿州是全省唯一被国务院评为“推广PPP模式工作有力、社会资本参与度较高的市”。创新能力进一步提升，发明专利授权量增长140.9%，研发经费投入增长27.9%，高新技术企业增至90家，建成省级孵化器1家、省级众创空间5家，埇桥区被评为全国科技创新百强区。首次成功举办全省青少年科技创新大赛。落实招商引资“1+5”配套文件，实际到位内资、利用外资分别增长10.7%、16.9%。加快推进与徐州等地互联互通，积极参加中国国际进口博览会、2018中国国际徽商大会、世界制造业大会等经贸活动，外贸进出口总额增长28.3%，对外开放的大门越开越大。

六是突出落细落实，民生福祉保障有力。坚持共享发展理念，民生保障力度持续加大，财政民生支出347.1亿元、占财政总支出的87.5%，33项民生工程全面完成。深化教育改革，落实教师待遇。推进义务教育均衡发展，新建、改扩建公办幼儿园64所，实施“全面改薄”项目建设校舍、维修改造农村中小学校舍共75.5万平方米，农村义务教育学生营养改善计划惠及学生30万人，新汴河景区被教育部命名为“全国中小学生研学实践教育基地”。组织参加安徽省第十四届运动会，金牌总数全省第八、获竞技体育贡献奖，在省第七届残疾人运动会上奖牌总数全省第四，在省第八届少数民族传统体育运动会上金牌数并列全省第二，王春雨勇夺2018雅加达亚运会田径女子800米金牌。深入开展全民健身活动，城市社区15分钟健身圈逐步形成。加快完善医疗卫生服务体系，持续深化综合医改，实施“四型医院”建设，4家二级医院开展三级医院创建；持续实施医疗卫生服务体系“411”计划，3家县级医院投入使用，新建、扩建乡镇卫生院20个，提前两年完成新建400个村卫生室的目标任务；启动卫生健康人才和专科建设“511”计划，创建省级重点专科10个；重大疾病防控成效显著，成功承办皖苏鲁豫四省联防联控合作机制会议暨突发公共卫生事件联合应急演练。实施更加积极的就业创业政策，全市城镇新增就业5.3万人，城镇登记失业率2.63%。完善社会保障体系，深入推进全民参保计划，五险参保人数190万人，新增养老床位2000张。主城区菜市场改造初见成效。困难群众社会救助工作受到民政部、财政部通报表扬，培育发展养老健康产业特色优势明显并获得省政府通报表彰。积极开展“宿州好人”“道德模范”系列评选活动，获评“中国好人”10人、“安徽好人”15人，砀山李娟荣获“全国五四青年奖章”。宿州荣获“文学之乡”称号。完善社会治安防控体系，强力开展“扫黑除恶”专项斗争，摧毁涉黑、涉恶犯罪团伙75个，“平安宿州”建设扎实推进。实施“深重促”专项行动，省交办我市的“2+3+X”信访件基本化解到位。食品药品安全工作持续加强，荣获“安徽省食品药品安全城市”称号。应急管理体系不断完善，安全生产形势持续向好，社会大局和谐稳定。“七五”普法活动扎实开展。第四次全国经济普查深入推进。双拥共建、人民防空成绩显著。民族宗教、防震减灾、外事侨务、物价、对台、气象、档案、地方志、妇女儿童、残疾人等事业不断进步。

在聚力经济社会发展的同时，我们始终高度重视政府自身建设。深入推进“两学一做”学习教育常态化、制度化，系统开展学习贯彻习近平新时代中国特色社会主义思想“五大一新”活动，扎实开展“讲忠诚、严纪律、立政德”专题警示教育。大力推进法治政府建设，依法全面履行政府职能，修订完善市政府工作规则，严格实施政府权力运行监管、重大事项合法性审查、政府法律顾问等制度。自觉接受人大及其常委会监督、政协民主监督和社会各界监督，全年办理人大代表议案和建议173件、政协委员提案298件。畅通群众联系渠道，12345政府热线服务平台受理群众诉求近13万件，办结率99%以上。灵璧县在全国基层政务公开标准化规范化试点推进会上作经验介绍。着力推进政府系统党风廉政建设，严管公共资金、强化审计监督、规范资源交易，锲而不舍加强作风建设，全面落实中央八项规定精神和省市实施细则，开展形式主义官僚主义问题大排查大调研，加强对侵害群众利益的不正之风和腐败问题专项整治，营造风清气正的良好环境，凝聚干事创业的磅礴力量。

各位代表！过去一年，我们爬坡过坎、负重前行，抗击了百年不遇的特大暴雨灾害，经受了多种风险挑战的考验；过去一年，我们求真务实、砥砺奋进，走过了一段艰苦卓绝的发展历程。经过这一年，全市广大干群的凝聚力、向心力、创造力显著增强，经济社会发展的协调性、持续性、抗压性显著增强，人民群众的获得感、归属感、自豪感显著增强，宿州对外的参与度、美誉度、影响力显著增强，展现了吃苦耐劳、坚韧顽强的精神禀赋，彰显出愈挫愈奋、越战越勇的“宿州特质”。全市上下在习近平新时代中国特色社会主义思想和党的十九大精神科学指引下，在省委、省政府和市委的坚强领导下，树牢“四个意识”，坚定“四个自信”，强化“两个维护”，立足实际，迎难而上，以百折不挠的坚强斗志和志在必胜的充分自信，实现了本届政府的良好开局。在此，我代表市人民政府，向奋斗在各行各业的全体劳动者，向离退休老同志，向各民主党派、工商联和人民团体，向中央和省驻宿单位、人民解放军驻宿部队、武警官兵、公安干警，向参与宿州建设的海内外企业家和客商、广大援宿干部，以及支持宿州发展的各界人士，表示衷心的感谢，致以崇高的敬意！

二、2019年重点工作

各位代表！今年是新中国成立70周年、全面建成小康社会关键之年，也是宿州市撤地建市20周年、脱贫退出巩固提升年、污染防治攻坚突围年。做好今年的工作，意义重大，任务艰巨，使命光荣。我们必须以坚如磐石的信念、战无不胜的信心、多谋善断的智慧、开放包容的胸怀，抢抓机遇、只争朝夕，全力推动经济社会高质量发展。

知难方能行易，行稳方能致远。我们必须清醒地看到，当前经济运行稳中有变、变中有忧、危机并存。一方面，国际环境复杂多变，国内深层次结构性矛盾和问题在外部冲击下趋于显性化，中美经贸摩擦是当前和今后一个时期我们面临的最大不确定因素，对经济运行多领域的传导效应不可低估。另一方面，我国经济由高速增长阶段转向高质量发展阶段，发展不平衡不充分问题和各种周期性、结构性、体制性因素交织叠加在一起。我市经济正处在结构调整的过关期，产业发展层次不高，实体经济困难较多，新的动能仍显不足；生态环境压力巨大，脱贫攻坚任务繁重，公共服务存在短板；部分干部责任担当不够，应对复杂问题能力不强，绣花功夫抓落实的作风有待提升。这都需要我们增强忧患意识，把困难估计得更充分一些，把措施考虑得更周密一些，把工作做得更扎实一些。

从有利因素看，国家和省围绕实施供给侧结构性改革、部署“六稳”工作、推进淮河生态经济带建设，释放出支持有效投资增长和新动能成长的积极信号，基础设施、乡村振兴、新兴产业等领域补短板力度将进一步加大，简政、减税、降费将在更大程度更大规模上进一步深化，财税、金融、投资等重点领域改革将进一步深入。我们要抢抓政策机遇，有针对性地抓重点、补短板、强弱项。总之，我市经济持续健康发展的基本面没有改变，高质量发展的积极因素仍在持续累积，延续经济平稳增长的向好态势大有空间、大有可为，我们完全有条件、有能力、有信心在新时代实现高质量发展。

今年工作的总体要求是：坚定不移地以习近平新时代中国特色社会主义思想为指导，深入贯彻落实党的十九大和十九届二中、三中全会精神，统筹推进“五位一体”总体布局，协调推进“四个全面”战略布局，坚持把发展作为第一要务，坚持稳中求进工作总基调，坚持推进高质量发展，坚持以供给侧结构性改革为主线，按照市委“2+4+2”工作总体要求，以五大发展行动计划为总抓手，继续打好三大攻坚战，扎实做好稳就业、稳金融、稳外贸、稳外资、稳投资、稳预期工作，保持经济持续健康发展和社会大局稳定，为全面建成小康社会收官打下决定性基础，以优异成绩庆祝中华人民共和国成立70周年。

综合考虑，今年经济社会发展主要预期目标是：全市生产总值增长8%以上，财政收入增长高于GDP增速，固定资产投资增长10.5%左右，社会消费品零售总额增长11%左右，城镇、农村常住居民人均可支配收入分别增长8%、9%左右，城镇调查失业率控制在5%以内，生态环境持续改善，节能减排完成年度任务，主要动态指标继续位居全省前列，完成全年各项目标任务。

重点抓好以下七个方面工作：

（一）聚焦产业转型，培育高质量发展新动能。坚决贯彻“巩固、增强、提升、畅通”八字方针，坚持把着力点放在实体经济上，不断优化产业结构，培育壮大经济发展新动能，为高质量发展提供持久强劲动力。

持续主攻先进制造业。深入推进“三重一创”建设，落实省重大新兴产业基地高质量建设方案，做强做优“1+9”省市两级战略性新兴产业基地，做大做强云计算、大数据、智能制造、石墨烯等新兴产业，加快推进生物医药加工重大工程建设，谋划培育MEMS传感器芯片制造产业，规上工业增加值增长9%，战略性新兴产业产值、高新技术产业增加值分别增长20%、13%。研究对接“数字江淮”建设指导意见，加快发展智慧经济，大力建设5G试验网络。深入实施重点技改“1525”行动计划，推动轻纺鞋服、板材家居等传统优势产业数字化、智能化转型，滚动实施重点技改项目200个，技改投资增长8%。紧盯重点领域、重点企业实施精准招商、集群招商，实际到位内资增长10%，实际利用外资增长6%。聚焦“1+2+N”目标，落实“四督四保”制度和项目工作五项机制，加大基础设施补短板和产业转型升级力度，开展高质量发展项目建设推进活动，重点项目储备规模2500亿元以上，实施省亿元以上重点项目超600个。大力开展质量提升行动，努力创建全国质量强市示范城市。

加快发展现代服务业。扎实开展扩消费专项行动，积极推进服务业集聚发展，服务业增加值增长10%。大力发展现代物流、健康养老、文化旅游等现代服务业，促进跨境电商综合服务平台建设，推进电子商务进农村全覆盖提质增效，支持灵璧电商创业特色小镇等重点项目建设。推进全域旅游发展，大力实施旅游强市“五个一批”建设工程，着力打造“一带两廊”三大旅游主轴，支持新汴河景区创建5A级景区。

着力促进区域协同发展。落实促进县域经济高质量发展意见，大力创建省级特色产业集聚发展基地，积极争取省级县域经济高质量发展专项引导资金，鼓励社会资本在县域发起设立招商联合基金。持续实施“3111”工程，推动各县区、园区首位产业全链条发展。全力建设“中国云都”“中部鞋都”，加快绿地科技产业园、宿州经开区生物医药科技园等重点项目建设，提升张江萧县高科技园、泗涂现代产业园等县域开发园区发展水平。促进园区改革创新发展和优化整合，加快推进市场化运营改革，加强园区污水处理厂、集中供热供气、标准化厂房等生产生活配套设施建设，持续增强承载能力。

全力支持民营经济发展。坚持“两个毫不动摇”，推动省促进民营经济发展“30条”各项政策举措落地见效，全力帮助民营企业融化市场的冰山、削平融资的高山、跨越转型的火山，让民营经济创造活力充分迸发。大力培育“专精特新”中小企业，持续壮大龙头骨干企业，着力支持民营企业创新发展，切实增强企业核心竞争力。全面落实国家、省各项减税降费政策，以及降成本“20条”等惠企利企政策，实质性降低企业制度性交易、用地用能用工等成本。持续强化精准金融服务，设立民营企业纾困救助基金，充分发挥各类产业基金的引导作用，引导金融机构扩大对民营企业、小微企业的信贷投放，着力解决民营企业融资难融资贵问题，确保民营企业发展不缺血、不断血。推动“非禁即入”普遍落实，进一步规范涉企执法行为，营造公平竞争环境。强化“四送一服”双千工程对民营企业的精准服务，健全政企沟通长效机制，组织实施“企业家培训计划”，维护民营企业合法权益，全力支持民营企业走向更加广阔的舞台。

（二）聚焦脱贫退出，筑牢全面小康社会奠基石。深入学习贯彻习近平总书记关于扶贫工作的重要论述，围绕“1234+N”工作思路，深入实施打赢脱贫攻坚战三年行动，因地制宜综合施策，确保实现5.33万贫困人口脱贫，萧县高质量脱贫退出，三县一区进一步巩固脱贫成果。

在夯实基层基础上聚合力。全面夯实贫困人口精准识别、贫困数据精确管理基础。强化扶贫对象动态管理，及时落实返贫户和新增贫困户帮扶措施。进一步健全“单位帮扶、干部驻村、整村包保”制度，持续推进县域结对帮扶，推动民营企业“百企帮百村”精准扶贫行动提质增效，广泛引导和动员各类社会组织参与脱贫攻坚。充分发挥基层党组织战斗堡垒作用，打造过硬的脱贫攻坚一线队伍。

在提升脱贫质量上再加力。全面落实革命老区脱贫攻坚各项政策举措，壮大村级集体经济，拓展深化“四带一自”产业扶贫、“三有一网”点位扶贫、“三业一岗”就业扶贫模式，大力推广“一自三合”扶贫小额信贷模式，强化光伏扶贫项目运维和收益分配监管，促进贫困村、贫困户持续稳定增收。全面落实教育扶贫政策，精准落实“351”“180”健康脱贫政策，持续推进危房改造工程，加强易地扶贫搬迁户后续帮扶，深入实施生态扶贫工程，不断完善兜底保障举措，打通政策落地“最后一米”。进一步巩固提升农村道路扩面延伸、安全饮水、农村电网、信息网络等基础设施建设，加快补齐“双基”短板。

在聚焦深度贫困上强攻坚。强化对萧县和全市8个深度贫困村的精准支持，持续加大政策、资金、项目、人力等各种资源要素支持力度，确保深度贫困地区如期实现脱贫目标。加快老汪湖行蓄洪区脱贫攻坚步伐，重点解决好安全、环境、发展问题，持续提升基本公共服务能力。全面落实特殊贫困群体脱贫攻坚政策，破解脱贫与解困两个难题。

在巩固脱贫成效上出实招。坚持扶贫与扶志、扶智相结合，建立有效防范返贫机制，科学制定实施后续帮扶计划和巩固提升方案，坚持脱贫不脱责任、不脱政策、不脱帮扶、不脱监管，做到脱贫攻坚工作力度、资金投入、政策支持、帮扶力度“四个只增不减”。持续加强扶贫领域腐败和作风问题专项治理，对已实现巡察全覆盖的贫困村适时开展“回头看”。健全实现稳定脱贫、夯实乡村振兴基础的长效机制，推进脱贫攻坚与乡村振兴战略有效衔接。

（三）聚焦污染防治，打赢生态环境保护翻身仗。深入贯彻习近平生态文明思想，制定实施“1+3”污染防治方案，打好标志性的重大战役，不断增强宿州人民生态获得感。

打赢打好“三大保卫战”。坚决打赢蓝天保卫战。全力推进蓝天保卫战三年行动计划，巩固提升“洁净宿州”专项行动。加强工业企业大气污染综合治理，整治完成“散乱污”企业及集群。大力推进散煤治理和煤炭消费减量替代。推进柴油货车污染治理，完善机动车遥感监测网络。深化道路扬尘污染防治，抓好露天矿山综合整治，加强餐饮油烟管控，持续强化烟花爆竹禁售禁放。继续做好秸秆禁烧工作，提升农作物秸秆综合利用率。推进大气测量基站建设，有效应对重污染天气，确保完成年度空气质量目标。着力打好碧水保卫战。持续推进主城区污水治理三年攻坚行动，加大住宅小区雨污分流改造力度，提升黑臭水体治理成效。加强河流综合治理，推动污水处理设施、地表水国考断面水质自动监测站、备用水源建设。持续加强农业农村污染治理，全市畜禽粪污综合利用率达到75%以上。扎实推进净土保卫战。强化土壤污染管控和修复，巩固2018“清废行动”整治成果，加快推进垃圾分类处理，狠抓固体废物污染防治，有效管控农用地和城市建设用地土壤环境风险。

加快转变生产生活方式。大力发展节能环保、清洁生产、清洁能源产业，在建材、化工、造纸、农副食品加工等行业全面推行清洁生产改造或清洁化改造。强化能源和水资源消耗、建设用地等总量和强度双控行动，单位GDP能耗降低和能源消耗总量完成省下达目标任务。启动国家公交都市创建活动，大力发展公共交通，加快新能源汽车发展推广。开展创建绿色家庭、绿色学校、绿色社区、绿色商场、绿色餐馆等行动，引导公众绿色生活。

常态长效推进环境保护。落实《宿州市城市生态网络规划》，制定实施全市生态系统保护和修复方案，统筹推进山水林田湖草生态保护与修复工程，划定并严守生态保护红线。坚持“一河一策”“一湖一策”“一林一策”，推深做实河长制、湖长制、林长制。建立健全生态环境保护法治体系、经济政策体系、能力保障体系、社会行动体系，建立常态化资金投入机制，制定落实污染防治攻坚战成效考核实施细则，推进生态环境损害赔偿制度改革，做细做实环境监管“网格化”，建立环境污染“黑名单”制度，持续巩固生态环境治理成效。增强服务意识，精准帮助企业制定环境治理解决方案。强力推进中央、省环保督察反馈问题整改，做到即查即改，确保改彻底、改到位。

（四）聚焦文明创建，推动城市功能品质大提升。围绕打造省际交汇区域中心城市，统筹推进城乡一体化建设，巩固提升文明创建成果，不断满足人民群众对大美宿州的向往。

增强城市承载能力。完善城市规划体系，全面推进“多规合一”，完成修订《宿州市城市总体规划（2012－2030）》《宿州市空间规划》，强化土地利用总体规划整体控制作用。突出亮化、绿化、美化，加快汴北新区、城东新区建设，主城区实施四大类130项重点工程，总投资194.03亿元，着力实现“双95”目标。深入推进“两治三改”专项行动，快速稳妥推进棚户区改造，加快安置房建设进度，实施和谐征迁、生态征迁。力促民航机场、淮宿蚌城际铁路、合新高铁泗县段、徐宿蚌高速公路、怀洪新河水系洼地治理工程、宿州船闸工程等重大工程建设，加快银河一路东西延伸工程、唐河路东延工程、宿州火车站综合改造、埇桥采煤沉陷区综合治理等项目建设步伐，建成使用“五馆一院一中心”，打通主城区、各园区断头路。加快推进污水管网、停车场、菜市场、充电桩等基础设施建设，大力构建“三环链城、四带穿城、五苑抱城、百园融城”的绿带体系。

统筹城乡联动发展。着力打造四个县城副中心，加快培育萧砀、灵泗小城镇集群，高品质推动省级特色小镇建设。大力推动100项县区重点工程建设，持续实施“555”工程，着力补齐城乡基础设施和公共服务设施短板。创新金融服务县域发展模式。深化户籍制度改革，积极引导农民进城落户，加大对农业转移人口市民化财政支持力度，推进已经在城镇就业的农业转移人口落户。

丰富城市发展内涵。加强文明城市创建，对照新版全国文明城市测评体系，紧扣“治乱、补缺、提升、固化、美化”等环节，深入开展“十项提升”行动，推进22项系列整治提升活动，全面整治小区物业管理、出店占道经营、乱搭乱建等突出问题。深入宣传践行《宿州文明20条》，扎实开展“道德模范”“宿州好人”评选活动，深化文明单位、文明行业、文明社区评选等系列创建活动，用好新时代文明实践中心这一有效平台，持续提升市民文明素养。延续城市历史文脉，打造宿州城市精神。深化城市执法体制改革，推进智慧城市建设，统筹实施智慧社区、智慧城管、智慧交通、智慧消防等项目建设，提高城市管理科学化、精细化、智能化水平。

（五）聚焦乡村振兴，促进农业农村现代化实现新突破。制定落实乡村振兴战略发展规划，扎实推进农业供给侧结构性改革，用好“两区一园”三个国家级平台，加快由农业大市向农业强市迈进步伐。

大力发展现代农业。深入实施新型农业经营主体培育工程，促进一二三产业融合发展，现代农业产业化联合体发展到295家，新增市级以上示范家庭农场、农民专业合作社示范社各50家，实现全市规模以上农产品加工产值占全省总产值9%以上。高水平建设农产品精深加工产业园，招大、扶强、奖优农业产业化龙头企业。大力发展乡村旅游和休闲农业，推进农业、林业、水利与旅游、文化、体育、康养等产业深度融合。强化农业资源环境管控、秸秆和畜禽粪污资源化利用、农业生态补贴，推进农业绿色发展。深入实施“优质粮食”“放心粮油”“主食厨房”工程，进一步优化粮食种植结构，保障粮食生产安全。实施质量兴农战略，推进“互联网+”在农业领域广泛应用，大力发展“三品一标”农产品，新增市级以上标准果园、蔬菜标准园各5个，着力增加优质绿色农产品供给。

加快建设美丽乡村。促进美丽乡村建设提质、扩面、连片，打造一批美丽乡村示范点。大力推进“四好农村路”建设，着力优化农村路网布局，加大县乡道路升级改造力度，加速城乡交通运输一体化进程。深入实施农村人居环境整治三年行动，持续开展农村环境“三大革命”，全面改善农村人居环境。加快推进高标准基本农田建设，大力实施农田水利“最后一公里”建设行动计划，巩固提升淮水北调及市级配水工程，加强自然灾害防治能力建设。开展移风易俗行动，加强农村公共文化服务基础设施建设，健全自治、法治、德治相结合的乡村治理体系。

深化农村综合改革。开展城乡建设用地“增减挂提质年”活动，加强增减挂节余指标市内调剂，提前谋划安置区建设，完成拆旧区先行复垦3万亩。加强农村土地制度改革，落实农村土地承包关系稳定并长久不变政策，完善农村承包地“三权分置”制度，积极推进房地一体的农村集体建设用地和宅基地使用权确权登记颁证，做好泗县国家农村集体产权制度改革试点工作。深化农村“三变”改革，加快集体经营性资产股份合作制改革，全面推进供销社综合改革。大力培育新型职业农民，扶持培育一批农业职业经理人、乡村工匠。进一步推进“劝耕贷”工作，建立健全适合农业农村特点的农村金融体系和多层次农业保险体系，提升农业抗风险能力。持续拓宽农民增收渠道。

（六）聚焦体制创新，全面提升改革开放外向度。以改革开放40周年为契机，深化改革创新加快动能转换，着力厚植开放型经济新优势。

重精准防范化解重大风险。深化政府隐性债务“四清四实”专项整治，规范政府举债融资机制，积极谋划争取专项债券资金，强化对融资平台、产业投资基金的规范化管理，妥善化解政府债务风险。加大不良贷款处置核销力度，持续整治互联网金融，深入开展防范和处置非法集资工作，建立健全地方金融监管体系，坚决守住不发生系统性金融风险底线。运用市场化、法治化手段稳妥处置“僵尸企业”，完成淘汰落后产能任务。

大力度推进重点领域改革。持续深化“放管服”改革，健全政府“3+2”清单制度体系，全面推开“证照分离”改革，落实“马上办、网上办、就近办、一次办”等各项便民利企举措，重点推动所有审批服务一网通办，最大限度激发市场活力。健全“政府承诺+社会监督+失信问责”机制，提升信用体系建设水平。深化科技体制改革，推进“双创”平台发展，发挥企业创新主体作用，加强知识产权保护和运用，加速推进科技成果转化。完善投融资体制，鼓励和支持社会资本参与基础设施、生态环保等重点领域建设，规范推进PPP项目建设。以金融体系结构调整优化为重点深化金融体制改革，新增贷款300亿元，力争推动主板上市企业1家，新增新三板挂牌企业2家。深化国资国企改革，完成市属国有企业公司制改制。持续推进34项国家级改革试点和34项省级改革试点，打造22项市级改革试验基地，推动财税体制、预算绩效管理、文化体制、统计体制、公共资源交易管理等重点领域改革落地见效。

全方位实施双向开放。加快融入“一带一路”和长江经济带建设，主动衔接长三角一体化发展，制定落实淮河生态经济带发展规划实施意见，加强与徐州等周边城市多维融合，谋划推进宿徐产业园建设，促进淮海经济区协同发展。推动大通道、大平台、大通关建设，强化基础设施互联互通，加快实施新汴河航道整治、浍河航道升级改造等重点工程，积极申报建设保税物流中心，大力建设内陆无水港，进出口总额增速高于全省平均水平。突出引智引技，深入推进人才强市“十百千万”提升工程，积极探索人才“团队+产业+园中园”的引才模式，打造皖北人才新高地。

（七）聚焦民计民生，建设文明共享新宿州。牢固树立以人民为中心的发展思想，以“七有”为重点大力实施民生工程，持续推进基本公共服务均等化，更好保障和改善民生。

织牢社会保障网。完善积极就业政策，全面实施“创业江淮”计划，推进新时代产业工人队伍建设，落实农民工返乡就业创业支持政策，加强企业职工培训再就业，促进高校毕业生、农民工、退役军人等群体就业创业，城镇新增就业3.1万人。大力推进全民参保计划，扎实推进城镇居民大病保险、城乡居民基本养老保险等民生工程，五险参保190万人次。完善城乡社会救助体系，健全农村留守儿童和妇女、老年人关爱服务体系，发展残疾人事业，加强残疾康复服务。

织紧公共安全网。大力实施安全发展战略，扎实推进城市安全发展，深入实施“铸安”行动，着力深化安全风险管控、隐患排查治理、安全专项整治，压实压紧安全生产属地监管、部门监管责任和企业主体责任，保持安全生产形势持续稳定。强化食品药品安全日常监管，积极争创国家级食品安全城市。深入开展“守护平安”系列行动，切实抓好“雪亮工程”建设，依法严厉打击盗抢骗、黄赌毒、网络诈骗等违法犯罪活动，纵深推进“扫黑除恶”专项斗争，启动实施社会心理服务体系建设试点工作，持续提升群众安全感。巩固“深重促”专项行动成果，完善矛盾纠纷多元化解和风险防控机制。增强应急联动和防灾减灾救灾能力。

织密公共服务网。全面深化教育综合改革，大力推进义务教育优质均衡发展，继续实施学前教育第三期行动计划；加大城乡中小学规划建设力度，加强教育集团化办学，提升教育资源供给能力；统筹推动普职协调发展、中高职衔接发展，推动校企合作、产教融合，加快构建现代职教体系，着力推动优质学科专业与高水平大学建设。加强教师队伍建设，全面整治校园周边环境，不断提高教育教学质量。持续推进健康宿州建设，深化综合医改，加快推进分级诊疗制度建设。加强全市“2+5”医联体、县区“1+4”医共体建设，扎实开展“四型医院”建设，强力推进医疗卫生服务体系“411”计划，稳步实施卫生健康人才和专科建设“511”计划，积极创建省级卫生城市，全面提升公共卫生服务水平。支持健康产业融合发展，加快老龄事业和产业发展，完善养老护理体系。大力建设体育强市，完善市体育馆功能，加快市、县区体育设施“五个一”建设，加强全民健身场地设施建设，办好宿州市第六届运动会。加快发展住房租赁市场，完善促进房地产市场健康发展长效机制。深入推进文化强市建设，推动文化事业和产业发展，加强文化遗产传承、保护与利用，加快县区图书馆、文化馆等场馆建设，开展重点文艺创作和戏曲大舞台、文化惠民消费季等群众性文化活动，扶持书画、戏曲等文艺事业发展。继续实施科普为民惠民行动，进一步提升全民科学素质。提升国防动员和后备力量建设水平，加强人民防空建设，实施军民融合深度发展行动计划，健全军民融合工作发展机制。扎实开展“七五”普法工作，持续推进“谁执法谁普法”责任制落实。完成第四次全国经济普查基本工作，筹备第七次全国人口普查。支持人民团体发挥桥梁纽带作用，加强民族宗教、外事侨务、广播电视、防震减灾、妇女儿童、红十字、地方志、档案、对台、气象等工作。

三、加强政府自身建设

使命呼唤担当，目标引领行动。我们将树牢“四个意识”，强化“两个维护”，增强服务群众、服务企业、引领发展能力，不断提升政府治理能力现代化水平，建设人民满意的创新政府、担当政府、法治政府、廉洁政府。

（一）深化改革，优化职能，提升政府系统的创造力。深入贯彻落实中央、省市委改革部署，以治理体系和治理能力现代化为导向，以推进机构职能优化协同高效为着力点，改革机构设置，优化职能配置，加快政府职能转变。加强和完善政府经济调节、市场监管、社会管理、公共服务、生态环境保护等职能，确保3月底前基本完成市县机构改革，构建系统完备、科学规范、运行高效的机构职能体系，再创体制机制新优势。

（二）强化效能，真抓实干，提升善作善成的执行力。牢牢锁定“保八争六”目标，抓主抓重抓关键，张弛有度“弹钢琴”，雷厉风行保落实。增强答卷意识、坚持目标导向，对每项工作实行任务菜单式分解、责任契约式落实、绩效审计式验收、结果问责式考核，完善保障工作落实的闭环链条。进一步统筹改进和规范考核监督机制，优化政府目标管理绩效考核体系，提高各类督查检查考核的质量和效率，切实减轻基层负担。健全正向激励、容错纠错机制，进一步激发干部崇尚实干、攻坚克难的责任担当，推动形成想作为、敢作为、善作为的良好风尚。把建设学习型政府摆在突出位置，全面增强“八种本领”，善于把握机遇、应对挑战、防控风险、处理矛盾，牢牢把握工作主动权。

（三）依法行政，权责法定，提升执政为民的公信力。严格遵守宪法和法律，深入开展法治宣传教育，着力提升法治思维、依法行政能力。提高政府立法实效，加强规范性文件制定管理。全面落实新修订的政府工作规则，推进政府决策科学化、民主化、法治化，提升行政决策公信力。深化综合执法改革，落实重大行政执法决定法制审核制度、行政执法公示制度，规范行政裁量行为，促进公正文明执法。依法接受市人大及其常委会工作监督和法律监督，自觉接受市政协民主监督、全力支持市政协履行职能，认真听取吸收各民主党派、工商联、无党派人士、人民团体和社会各界意见。创新群众工作体制机制和方式方法，主动接受社会监督、舆论监督，深入推进政务公开，发挥好12345政府热线平台作用，让权力公开透明、阳光运行。

（四）从严治政，廉洁从政，提升履职尽责的保障力。认真履行全面从严治党主体责任，持续推进“两学一做”学习教育常态化、制度化，巩固拓展“讲严立”专题警示教育成果，加强、规范党内政治生活，自觉加强和接受党内监督。深化廉政风险防控，严防权力滥用和设租寻租，加强对环境保护、精准脱贫、公共资源交易、国有资产管理等重点领域的监督管理。牢记作风建设永远在路上，深入贯彻落实中央八项规定精神及省市实施细则，驰而不息整治“四风”问题新形式新表现，严肃查处侵害群众利益的行为。支持纪检监察机关依法依规履行职责，推进审计管理体制改革和审计监督全覆盖，强化审计结果运用，推动干部清正、政府清廉、政治清明。

各位代表！幸福是奋斗出来的，美好宿州的未来就在我们手上。让我们更加紧密团结在以习近平同志为核心的党中央周围，高举习近平新时代中国特色社会主义思想伟大旗帜，在省委、省政府和市委坚强领导下，撸起袖子加油干，担当尽责创佳绩，奋力谱写全面建成小康社会、加快建设现代化五大发展美好宿州的壮丽篇章，以亮丽的成绩庆祝中华人民共和国成立70周年！
